# ARDS associated acute brain injury: from the lung to the brain

**DOI:** 10.1186/s40001-022-00780-2

**Published:** 2022-08-13

**Authors:** Mairi Ziaka, Aristomenis Exadaktylos

**Affiliations:** 1 Department of Internal Medicine, Thun General Hospital, Thun, Switzerland; 2 Department of Emergency Medicine, Inselspital, University Hospital, University of Bern, Bern, Switzerland

**Keywords:** Acute brain injury, Acute respiratory distress syndrome, Acute lung injury, Brain–lung interactions, Hypoxemia, Blood–brain barrier disruption, Inflammation, Mechanical ventilation

## Abstract

A complex interrelation between lung and brain in patients with acute lung injury (ALI) has been established by experimental and clinical studies during the last decades. Although, acute brain injury represents one of the most common insufficiencies in patients with ALI and acute respiratory distress syndrome (ARDS), the underlying pathophysiology of the observed crosstalk remains poorly understood due to its complexity. Specifically, it involves numerous pathophysiological parameters such as hypoxemia, neurological adverse events of lung protective ventilation, hypotension, disruption of the BBB, and neuroinflammation in such a manner that the brain of ARDS patients—especially hippocampus—becomes very vulnerable to develop secondary lung-mediated acute brain injury. A protective ventilator strategy could reduce or even minimize further systemic release of inflammatory mediators and thus maintain brain homeostasis. On the other hand, mechanical ventilation with low tidal volumes may lead to self-inflicted lung injury, hypercapnia and subsequent cerebral vasodilatation, increased cerebral blood flow, and intracranial hypertension. Therefore, by describing the pathophysiology of ARDS-associated acute brain injury we aim to highlight and discuss the possible influence of mechanical ventilation on ALI-associated acute brain injury.

## Introduction

Despite the remarkable advances in the management of acute lung injury (ALI) and acute respiratory syndrome (ARDS), ALI remains a relatively common and highly morbid or lethal condition [1]. ARDS-associated brain dysfunction is one of the most common complications in critically ill ARDS patients and represents one of the most frequent organ insufficiencies, often persisting months after hospital discharge [2]. Although the pathogenesis of ARDS associated brain injury is still not fully understood, it is well established that lung and brain represent an integrated ensemble [3], which interacts strongly through complex pathophysiological pathways [4]. Various factors have been suggested to contribute to the pathogenesis of ARDS-associated brain injury, including hypoxemia, neurological adverse events of lung protective ventilation, hypotension, disruption of the blood–brain barrier (BBB), and altered neurotransmission. Three main mechanisms seem to be involved to its manifestation, that is, inflammation, hypoxemia, and adverse events of mechanical ventilation [4–7]. Recognition and understanding of the pathophysiological mechanisms associated with ARDS encephalopathy might lead to improved clinical outcomes and therapeutic implications. The present review aims to analyze the various clinical central nervous system presentations in patients with ARDS. Moreover, we sought to describe the pathophysiology of neurologic manifestations, which occur secondary to ALI from a mechanistic standpoint. More specifically, we discuss the pathophysiological issues related to lung–brain interactions and provide an updated overview regarding the role of inflammation, hypoxemia, and haemodynamics on the development of ARDS-associated secondary brain injury. Finally, the impact of mechanical ventilation (MV) on the pathogenesis of acute brain injury in ARDS patients without preexisting brain injury is also analyzed.

## Clinical CNS presentations in ARDS patients

**Fig. 1:**
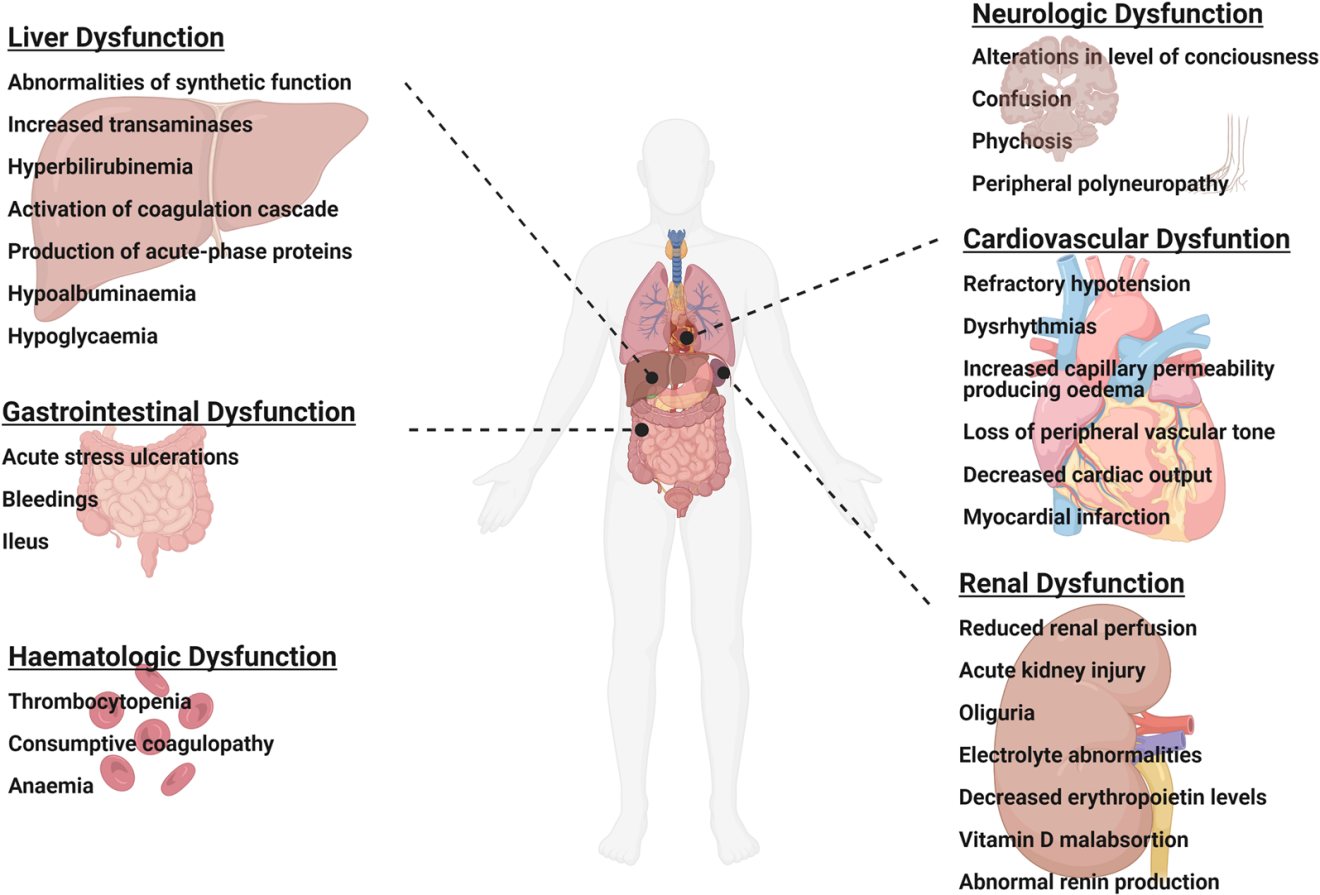
Extrapulmonal complications in patients with ARDS

Traditionally, refractory respiratory failure has been considered a relatively rare cause of death in patients with ARDS, occurring in about 20% of the patients [8–10], while studies report irreversible respiratory insufficiency in only 16% of ARDS patients [8]. However, the classical study of Ferring et al. [10] with 129 ARDS patients demonstrated sepsis, that is, multi-organ failure (MOF), as the primary mortality cause in 49% of ARDS patients, followed by respiratory failure (16%), cardiac dysfunction (15%), severe neurological injury (10%), and other causes (8%) (Fig. 1) [10]. ARDS is an acute inflammatory condition characterized by the release of pro-inflammatory mediators into the systemic circulation. The most important pro-inflammatory mediator are interleukin (IL)-6, IL-1β, IL-8 and tumor necrosis factor (TNF)-a, leading potentially to dysfunction of distant organs and systems [11–13]. The central nervous system appears to be one of the most targeted organs and systems [2]. Indeed, a study by Hoppkins and co-authors testing the assumption that ARDS may cause hypoxemia-induced brain injury found that all ARDS survivors (i.e., 100% of the cases) manifested cognitive and affective impairments at discharge. Interestingly, even 1 year after ARDS was observed, almost 80% of the patients still suffered from at least one neurocognitive alteration (i.e., impaired memory, attention, concentration, and/or mental processing speed) [14]. This first evidence were further supported by clinical and experimental studies showing that patients with ARDS seem to be at increased risk for developing Intensive Care Unit [15] delirium, independent of MV. Moreover, the majority of this group of patients appears to develop new cognitive, functional, and physical impairments with long-term consequences in their quality of life [16–20]. Recently, studies tracking the impact of coronavirus-2 (SARS-CoV-2) outbreak on critically ill patients described the emergence of a variety of neuropsychiatric features, such as encephalopathy, agitation, confusion, inattention, disorientation, and poorly organized movements in critically ill SARS-CoV-2 patients with severe acute respiratory failure [21–23].

Risk factors for the significant increase of the emergence of delirium include advanced age, preexisting neurocognitive disorders, history of alcohol abuse, severe systemic disease (e.g., sepsis and diseases of the respiratory tract), metabolic abnormalities, inadequate pain management, mechanical ventilation, surgery, and drugs [24]. Classic pharmaceutical agents implicated in the development of neurocognitive dysfunction include benzodiazepines, steroids, long-acting opioids, and anticholinergic medications, highlighting their use with great caution in elderly patients [25, 26]. Moreover, sedation and analgesia are frequently used to control anxiety and motoric unrest, pain and agitation, autonomic disability avoidance, brain metabolism reduction, and MV optimization [27, 28]. Furthermore, light rather than deep sedation and brief cessation of sedation for daily wake-up tests are recommended for reducing the risk of neurocognitive dysfunction, duration of MV, and hospital stay based on facts which show that depth of sedation may trigger the development of delirium [20, 26, 29–33].

ICU delirium is multifactorial with its pathophysiology comprising the influence of the underlying illness (e.g., sepsis, trauma, etc.), neuroinflammation, cerebral hypoperfusion from hypoxemia, breakdown of the BBB, disruptions of cerebral blood flow (CBF), and endothelial dysfunction [34]. On the other hand, however, critically ill patients have an additional contribution from MV; MV despite being lifesaving could as a side-effect exacerbate pulmonary and systemic inflammation and, hence, lead to lung and distant organ injury [4, 34]. In fact, it has been shown that critically ill patients are commonly admitted to the ICUs for MV [35]. Patient-ventilator interactions and the likelihood for developing acute lung injury is largely determined by the neural control of ventilation and the immune response, which could make patients susceptible to develop short- and long-term neuropsychological impairments, including delirium, sleep disturbances, persistent cognitive impairment, and post-traumatic stress disorder [36]. Although the interactions of the mechanically ventilated lung and CNS are complex and discussed in detail in the Pathophysiology Section, experimental and clinical studies suggest the existence of an interacting signalling. This interacting signalling could be due to a physiological mechanism, as for example the Hering–Breuer reflex. Another possible cause could be a pathological process, such as excessive alveolar stretch and over-distension, which could lead to maladaptive responses and contribute to the observed neurocognitive impairments and psychological alterations [36, 37].

Given that ICU delirium prolongs hospitalisation duration of mechanical ventilation patients and, ultimately, increases mortality [20, 30–32] emphasis should be given on prevention strategies and possible interventions, involving early identification of risk factors, on-time recognition of latent neurocognitive symptoms, avoidance of triggering factors, and multifactorial patient approach.

## Pathophysiology of ARDS-associated brain dysfunction

### The role of inflammation

More than two decades ago, Slutsky and Tremplay tried to explore the hypothesis of the contribution of lung injury and mechanical ventilation in the initiation of systemic inflammatory responses, which could lead to multiple systemic organ failure [38]. After their publication in 1997, an increasing body of evidence suggests that acute lung injuries and mechanical ventilation could elicit release of inflammatory mediators into the systemic circulation, creating a pro-inflammatory environment with potential detrimental effects on distal organs including the brain [39, 40]. The theory of systemic inflammatory reaction with subsequent deleterious effects on distant organs is further supported by anatomical and functional components. The pulmonary vasculature hosts up to one third of all neutrophils outside the bone marrow; as it receives the entire cardiac output, there is a significant potential for interaction with circulating neutrophils [38]. In addition, disruption of the lung microvascular barrier due to increased endothelial and epithelial permeability and alveolar injury due to high levels of pro-inflammatory cytokines allow efflux of inflammatory mediators into the systemic circulation [41].

Moreover, common causes of acute lung injury such as trauma, infections, or surgery not only affect the lungs but also generate further systemic inflammation and organ dysfunction through dysregulated production of inflammatory mediators, excessive endothelial dysfunction, alterations of the blood–brain barrier (BBB), neuroinflammation, and neuroglial cell death [42, 43]. Cytokines such as IL-1β and TNF-a are central mediators of neurogeneration [44, 45] suggesting a potential link between acute lung injury and development of brain dysfunction. Indeed, cytokines (e.g., TNF-a, IL-1a, IL-1b and IL-6) can directly cause neuronal apoptosis and produce a stereotyped cluster of nonspecific signs such as impaired concentration, anorexia, fatigue, diminished motivation, depression, and anorexia [46].

Interestingly, at the molecular level, hippocampus, which is involved in memory and learning processes, has a high density of IL-1 receptors, which could explain the common occurrences of hippocampal injuries in patients with acute lung injury regardless of the degree of hypoxia [47–49]. Experimental models of ARDS found a significant increase in S-100b protein levels—a plasmatic marker of brain injury—compared to hypoxic animals without ARDS [50]. Moreover, hippocampal damage occurred only in individuals with ARDS. Fries et al. concluded that for the same degree of hypoxemia, acute lung injury results to stronger brain injury when compared to hypoxemia induced by reducing the inspired oxygen fraction [50]. This suggestion is in accordance with the study of Nguyen et al. [51], who reported that elevated concentrations of S-100b and neuron-specific enolase (NSE) are frequently associated with brain injury in patients with severe sepsis and septic shock [51]. This pathogenetic mechanism is further supported by an experimental study in pigs with ARDS and intracranial hypertension, in which it was found that their combination induced damage in the hippocampus and decreased density in brain CT due to hypoxia-associated cerebral oedema, indicating synergistic exacerbation of pre-existing brain damage [52].

### Effects of inflammation in blood–brain barrier

The vascular BBB is an important neurobiological structure with a highly regulated interface between the blood and the brain [53]. It responds to signals of the immune system and regulates the neuroimmune communication of compartments of the blood and the brain [54]. However, during prolonged central nervous system inflammation [7] and systemic inflammatory response, a variety of soluble inflammatory mediators may influence the integrity of BBB and the subsequent neurological outcomes of the patients [53, 55]. Peripheral cytokine elevation activates supraphysiological responses, which alter the BBB, including increased solute permeability and lymphocyte trafficking, activation of endothelial cells, impairment of systemic and cerebral blood flow, and alterations of glucose metabolism in the brain [53, 56].

Experimental studies suggest that microglial cell activation plays a fundamental role in the development of BBB alterations [57, 58]. It has been also reported that enhanced peripheral cytokines induce a massive biochemical cascade that activates intracranial located microglia, the resident brain macrophages, to produce pro-inflammatory cytokines to recruit monocytes to the brain, leading ultimately to neuronal apoptosis and cerebral oedema [4, 59–61].

In addition, activation of the microvascular endothelial cells due to the binding of peripheral cytokines to the endothelium of vascular BBB alters adhesion and permeability and results to active cytokine transport between blood and brain compartments [61–63]. Indeed, elevated cytokines have been described in the CSF of critically ill patients, indicating disruption of the BBB and neuroinflammation [54, 64], findings further supported by recent studies in SARS-CoV-2 patients with neurological presentation [65–67]. Accordingly, previous clinical and experimental studies describe elevated plasma levels of S-100b and NSE, indicating BBB dysfunction, astrocyte and neuronal injury [37, 50, 51].

### Inflammation and mechanical ventilation

Mechanical ventilation is often a crucial life-support tool in the resuscitation of patients with acute lung injury and ARDS [68, 69]. However, mechanical ventilation per se can induce brain damage either by inducing an excessive release of pro-inflammatory cytokines (e.g., Il-1b, IL-6 and TNF-a) or by changing the vagal signal leading to neuroinflammation and neuronal death [37, 50, 68, 70–74]. Interestingly, an increased amount of evidence suggests that even a short period of mechanical ventilation may dramatically increase hippocampal and plasma levels of IL-1b, IL-6 and TNF-a [71]. This proposal is further supported by a recent experimental study of Sparrow and colleagues, who reported that acute lung injury due to mechanical ventilation induced reversible neuronal injury and inflammation in the frontal cortex and hippocampus of mechanically ventilated mice. Importantly, inhibition of IL-6 signalling reduced frontal and hippocampal apoptosis [75].

Clinical and experimental evidence have shown that the ventilator strategy may activate or even propagate the systemic inflammatory response, leading to dysfunction of multiple organs and systems, including the brain [38, 73]. Over the past 20 years, numerous studies have demonstrated a biological response with release of a variety of pro-inflammatory mediators and local initiation of inflammatory processes induced by an injurious ventilator strategy [69], which can potentially cause local lung injury. In addition, distal organs are also affected due to decompartmentalization of local inflammation to translocation into the systemic circulation [69]. The fact, that mechanical ventilation strategy using high tidal volumes may enhance the release of mediators such as IL-1b, IL-6 and TNF-a, is well recognized [76, 77]. Specifically, for the lung–brain crosstalk, it has been proposed that mechanical ventilation may propagate regional brain activation. For example, the experimental study of Quilez et al. showed that MV with high tidal volumes can cause more c-fos brain expression, a marker of brain activation, in healthy rats compared to protective mechanical ventilation, supporting the iatrogenous enhancement of neuroinflammation [74].

Although the mechanisms through which lung damage can reach the central nervous system are still poorly understood [74], the pivotal role of mechanotransduction (i.e., the conversion of mechanical stimuli into biological signals by mechanoreceptors) in this crosstalk has been proposed [76, 78]. Nowadays, it is also recognized that pro-inflammatory mediators can reach key structures in the brain via circumventricular organs and activate the autonomous nervous system in the periphery [79].

Hence, the occurrence of a lung–brain interplay through different mechanisms and biochemical pathways underlines the need for greater control of modified variables, as for example mechanical ventilation in the maintenance of lung and brain homeostasis.

## The role of hypoxemia

The brain is a highly metabolic and oxidative organ accounting about 20% of the basal oxygen budget, despite its small size, which represents about 2% of body weight [80]. Therefore, the brain is vulnerable to hypoxemic conditions [81]. Indeed, hypoxemia has been incriminated for causing tissue hypoxia and for increasing the risk of multiple organ failure including the brain [82]. However, although a number of studies have shown various pathogenetic effects of hypoxemia on the brain function of patients with severe respiratory insufficiency, it still remains unclear, if hypoxemia is a contributing cause for the emergence of cognitive dysfunction [81, 82]. Until now, it is well established that hypoxic stimuli can compensatory increase cerebral brain flow via cerebral vasodilation in order cerebral oxygen delivery to be maintained [81]. In addition, tissue oxygenation is regulated from various parameters, as for example dissolved oxygen, haemoglobin concentration, cardiac function, pH, and body temperature [81, 82]. In a clinical study of mechanically ventilated ALI-survivors, Mikkelsen et al. found that a mean PaO_2_ of 72 mmHg was significantly associated with long-term cognitive dysfunction in comparison to a mean PaO_2_ of 87 mmHg [83]. However, it should be kept in mind that hypoxic stimuli activate peripheral chemoreceptors, which could in turn lead to hyperventilation, subsequent hypocapnia and cerebral vasoconstriction, and reduced cerebral perfusion [84].

Cerebral microbleeds are small, hypodense lesions with a maximum size of up to 10 mm on haemorrhage sensitive MRI sequences. Although their causal association with chronic hypertension, cerebral amyloid angiopathy, and diffuse axonal injury is well documented, it has been recently noted that they may be causally related to less identifiable originators, such as sepsis and ARDS [85]. Riech and colleagues described in 2015 multiple microhaemorrhages, predominantly in the splenium of the corpus callosum, on the MRI of three patients who survived ARDS, findings that are typically seen in patients with high-altitude lesions, raising the question of common pathogenetic mechanisms between the two disease entities [86]. More recently, similar findings were presented by Fanou et al. in 12 patients with respiratory failure, of whom 11 received mechanical ventilatory support and 3 were on extracorporeal circulation. More specifically, the authors described haemorrhagic microlesions, diffusely involving the juxtacortical white matter and corpus callosum but sparing the cortex, deep and periventricular white matter, basal ganglia, and thalami [87]. These findings seem to be confirmed by more recent studies in patients affected with severe SARS-CoV-2-infection. Indeed, a recent meta-analysis showed that these patients exhibit much less deep microbleeds or lobular microbleeds, findings that are typically seen in patients with hypertensive angiopathy and cerebral amyloid angiopathy. In these patients a heterogeneous pattern of cerebral haemorrhagic manifestations is described, such as diffuse cerebral microhaemorrhages, affecting deep cortical white matter structures, including the corpus callosum as well as the brainstem and the cerebellum [88]. Although haemorrhagic manifestations are reported as relatively frequent complications in patients with ARDS (25%) [5], the exact pathogenetic mechanism for their occurrence still remains unclear. One hypothesis states that hypoxemia and inflammation could lead to endothelial and BBB dysfunction and, additionally, to extravasation of erythrocytes, resulting to diffuse cerebral microbleedings [87]—a phenotype of small vessel disease—which may further evolve to haemorrhagic stroke [5].

Ischaemic brain injury is another type of injury commonly observed in patients with acute lung injury. Its pathophysiologic mechanisms include activation of the endothelial cells and systemic inflammation, which result to subsequent activation of the coagulation system and to thrombi formation. Increased risk of ischaemic stroke is additionally associated to reactive oxygen radicals due to acute lung injury and hypoxemia [89], while prolonged hypoxemia in patients with severe respiratory failure leads to reduced delivery of oxygen and glucose to the brain. As a consequence mitochondrial dysfunction and upregulation of energy-dependent ion chains are manifested, causing neuronal apoptosis, necrosis, and cytotoxic oedema [5, 6]. This observation is of high importance especially in reference to structures, which are more sensitive to diffuse ischaemic injury due to their high metabolic demands (e.g., hippocampus and grey matter structures) [90] and is in accordance with the study of Janz et al., who reported that hypoxic brain injury in patients with ARDS was most commonly observed in the pyramidal neurons in the CA1 region of the hippocampus [49]. The pathophysiological mechanisms through which acute hypoxia results to hippocampal injury include glycolysis, increase of adenosine concentrations, cardiopulmonary compensatory response, oxidative stress, and mitochondrial disruption. These mechanisms end up ultimately to decreased synaptic plasticity, neuronal necrosis, and inhibition of long-term potentiation [91].

Furthermore, hypoxemia has been incriminated for the development of cerebral oedema and diffuse cerebral atrophy, although it still remains unclear, if the underlying responsible pathogenetic mechanism is hypoxemia or inflammation [5, 6].

Finally, it is demonstrated that erythropoietin [92], which is endogenous expressed in the CNS, is capable to induce neuroprotective properties in vivo and in vitro. EPO and EPO receptors are expressed in various brain regions and hypoxic/ischaemic insults predominantly stimulate their expression [93–99]. EPO- signalling plays a pivotal role in adult neurogenesis and neuroblast migration to ischaemic regions in vivo, besides its direct protection of neurons and modulation of the angiogenic response. It has been also shown that EPO expression is mainly restricted to some cellular types, predominantly astrocytes, but also neurons [93, 100, 101]. In addition, the hypoxia inducible factor [102]—a heterodimer of HIF-a and HIF-β subunits—has been found to regulate hypoxia-induced stimulation of EPO expression. In more detail, oxygen levels are effective inducers of the HIF-a subunit expression, whereas the expression of the HIF-β subunit is constitutive and dimerises with transcription factors. Interestingly, a key study examined the role of HIF-1a and HIF-2a (i.e., two of the three HIF-a subunits) in the generation of paracrine protective signals by astrocytes, which modulate the survival of neurons exposed to oxygen–glucose deprivation. The study showed that HIF-2a is the main regulator of EPO expression in astrocytes during hypoxia, indicating that astrocytes play an important neuroprotective role during hypoxia/ischaemia [103].

## Mechanical ventilation

**Fig. 2:**
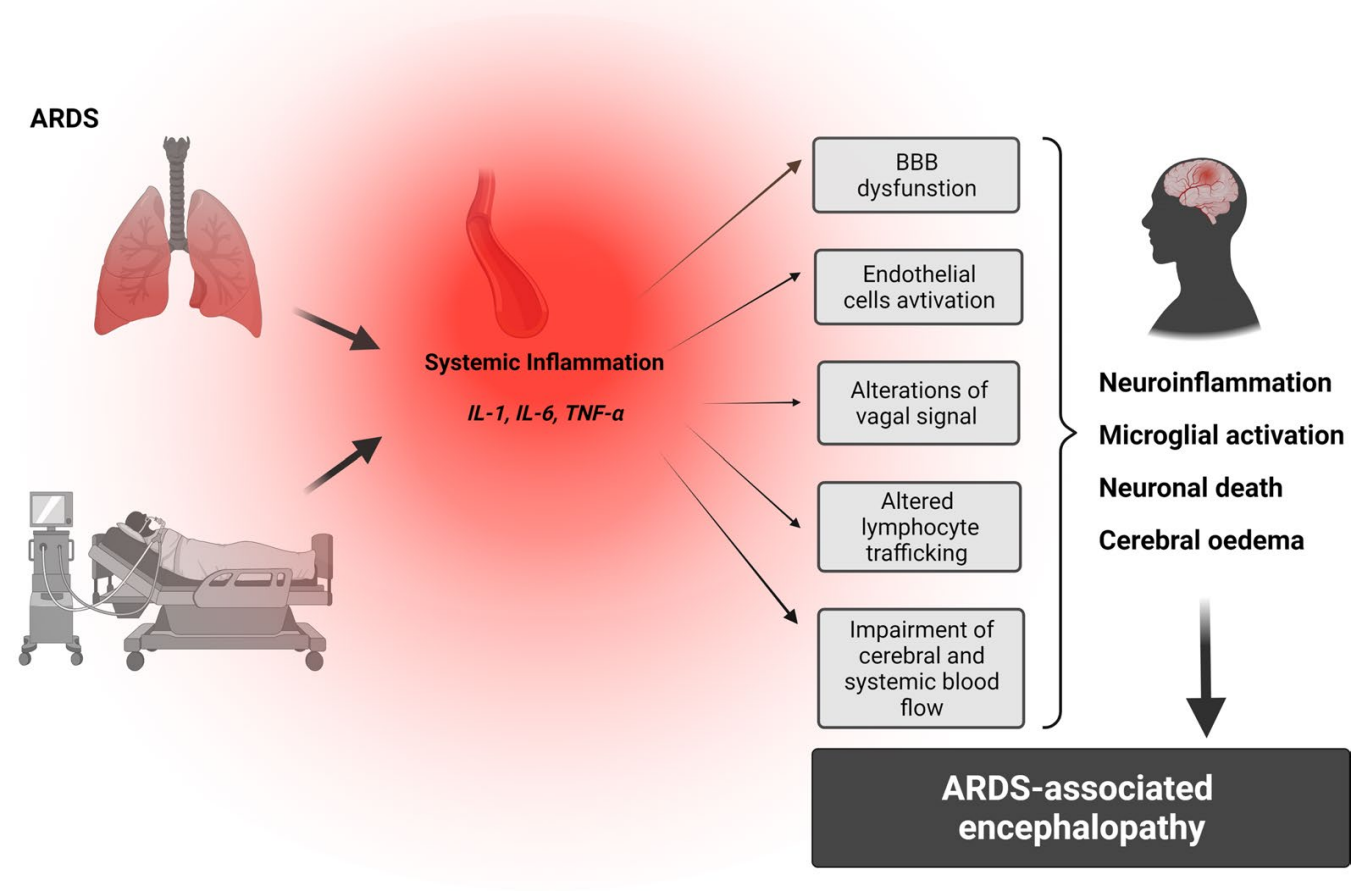
Role of inflammation in the development of ARDS-associated secondary brain injury. Hypoxemia and mechanical ventilation elicit a number of systemic responses including release of inflammatory mediators in the systemic circulation followed by potential BBB dysfunction, endothelial cell activation, altered lymphocyte trafficking, and impairment of cerebral and systemic blood flow. These afferent signals and circulating inflammatory mediators might induce neuroinflammation, microglial activation, neuronal death, and cerebral oedema, contributing potentially to the development of ARDS-associated encephalopathy

As mentioned above, although mechanical ventilation is a life-saving therapeutic intervention in the management of critically ill patients, it is well documented that it can trigger or exacerbate pulmonary and systemic inflammation [104, 105]. The underlying pathogenetic mechanisms include overstretching, recurrent alveolar collapse, and re-expansion during each respiratory cycle [106]. In addition, it appears that the conversion of mechanical to biological stimuli is involved in the pathophysiology of ventilator-associated lung injury [107] with deleterious effects both locally on the lung level and on distant organs and systems including the central nervous system [4, 108]. Multiple mechanisms, including neuroendocrine, inflammatory, hormonal and neural pathways, appear to be involved in mechanical ventilation-related brain damage [4, 105, 109]. In addition, it has been shown that an imbalance in neurotransmitters (i.e., dopamine and acetylcholine) contributes to the development of cognitive dysfunction in critically ill ICU patients [2, 49, 110, 111]. Previous research demonstrates that mechanical ventilation alters the vagal signal, leading to neuroinflammation and neuronal death (Fig. 2) [50, 70–74, 112–114]. Indeed, numerous studies have documented an increase in the concentration of inflammatory cells in the hippocampus mediated by the vagus nerve, affecting postoperative memory in experimental mouse models [71, 115]. The hypothesis of vagus nerve mediation in the induction of cerebral inflammatory response seems to be further supported by the finding that performing bilateral vagotomy prior to mechanical ventilation in mice protects against the development of brain damage [70]. In addition, several preclinical studies concluded that patients, who were mechanically ventilated for prolonged periods of time, showed deteriorated cognitive functions compared to patients, who were not mechanically ventilated, or patients, who received mechanical ventilation for a short period of time [71, 112, 113]. Furthermore, increased concentrations of inflammatory cells and proapoptotic proteins have been reported in the brains of patients receiving mechanical ventilation support [50, 70–74, 112–114]. Moreover, it appears that patients who received higher tidal volumes have more intense hippocampal activity, as shown using functional MRI, resulting in greater tissue damage than patients mechanically ventilated with lower tidal volumes [70]. Finally, it has been shown that higher tidal volumes may result in abnormal neuronal activity in the retrosplenial cortex and thalamus, as evidenced by higher c-Fos concentrations in these brain regions, compared to lower tidal volumes [74].

It is well documented that protective mechanical ventilation with low tidal volume and positive end-expiratory pressure (PEEP) in patients with ARDS improves outcome by reducing lung strain and preventing inflammation [4, 78]. However, it should be taken additionally into account that a protective ventilator strategy may lead to self-inflected lung injury, hypercapnia and subsequent cerebral vasodilatation, increased cerebral blood flow, and intracranial hypertension [116]. Nevertheless, the harmful of permissive hypercapnia was investigated in a small study of 12 patients with subarachnoid haemorrhage undergoing protective mechanical ventilation. The authors reported that mechanical ventilation with tidal volume of 5–8 ml/kg and moderate levels of PEEP led to PaCO2 levels of 50–60 mmHg without negatively affecting intracerebral pressure [117]. Moreover, clinical and experimental studies have shown that ventilation with high tidal volumes induces higher hippocampal activation associated with more tissue injury and a pathological neuronal activity, suggesting an iatrogenic effect of high tidal volume ventilation on the brain [68].

High PEEP is another part of the protective ventilation strategy used in ARDS to prevent alveolar collapse, recruit alveoli, and reduce atelectrauma [4, 118]. Yet, PEEP may also increase intracranial pressure (ICP), reduce cerebral venous return, and cerebrospinal fluid outflow [4, 119]. The underlying pathophysiologic mechanisms are complex and involve many factors among them cerebral vasodilatation due to elevated intrathoracic pressures and reduced mean arterial pressure [4, 119]. On the other hand, it has been suggested that PEEP increases ICP, only when PEEP causes alveolar hyperinflation, although when PEEP causes alveolar recruitment, there is no influence on cerebral perfusion and ICP [120].

Given the adverse effects of mechanical ventilation (e.g., the release of local and systemic inflammatory response, the often required deep sedation and the neuromuscular blockade and immobility), the alternative of avoiding it seems increasingly interesting [121]. Despite controversial and conflicting views, non-invasive ventilation (NIV) can be considered for the initial support of patients with ARDS [122, 123]. Indeed, the LUNG SAFE study showed that 15.5% of patients with ARDS initially underwent non-invasive mechanical ventilation. However, these patients were found to have lower PEEP levels and higher respiratory volumes and respiratory rates than patients receiving invasive mechanical ventilation. In addition, the use of NIV was associated with greater mask leaks, patients’ intolerance, and gastric distension [124]. In addition, it appears that the probability of failure of NIV increases significantly with the severity of ARDS [124], while at the same time this failure worsens the outcome [125], suggesting that delays in intubation can have devastating effects. On the other hand, it appears that the use of NIV as an initial approach in patients with ARDS is associated with avoidance of intubation in half of the patients and, thus, with a lower incidence of ventilator-associated pneumonia and related mortality [122, 126]. Due to conflicting evidence from existing clinical studies and the lack of well-documented recommendations for or against the use of NIV in severely affected patients with ARDS [127], its use should be limited to strictly selected patients. Further high quality research is needed to clearly define the role of NIV in the treatment of critically ill patients with ARDS.

## Haemodynamic compromise

Haemodynamic instability is a leading cause of increased mortality in patients with ARDS and is frequently associated with cor-pulmonale, deleterious effects of MV on the right ventricular function and the pulmonary vascular mechanics, being additionally related to sepsis [128, 129]. Specifically, mechanical ventilation causes changes in lung volume and, consequently, alters the vascular tone and the pulmonary vascular resistance. Especially when high tidal volumes are used, mechanical ventilation may lead to cardiac–tamponade similar phenomenon by compressing the heart in the cardiac fossa [130]. Moreover, changes in transpulmonary pressure influences right ventricular afterload, whereas alterations in pleural pressure affect venous return, leading to haemodynamic compromise [128].

Because the brain is an intensive metabolic organ, accounting for about 20% of the total body’s consumption of oxygen [131, 132] under normal conditions, CBF is approximately 50 ml/min/100 g of brain tissue and remains constant, if mean systemic arterial pressure ranges between 60 and 150 mmHg, ensuring brain’s autoregulation [133–135]. Following this reasoning, it may be concluded that haemodynamic instability in mechanically ventilated patients with ARDS impairs CNS homeostatic mechanisms, making the brain vulnerable to the development of secondary injury [128, 129, 136, 137].

A growing body of evidence supports the hypothesis that haemodynamic alterations, resulting to cerebral hypoperfusion, play a fundamental role in the development of neurocognitive dysfunction in critically ill patients [138, 139]. Experimental and clinical studies have shown that brain hypoperfusion is clearly associated with metabolic and energetic dysregulation, degeneration of brain capillaries, loss of cholinergic receptors, disruption of protein synthesis, and neuronal damage, affecting specific brain regions sensitive to the above mentioned processes and, predominantly, the hippocampus [91, 140, 141]. However and especially in patients with septic shock, microcirculatory changes along with macrocirculatory changes, may impair neurovascular uncoupling, disrupt the BBB, and activate the coagulation cascade, leading to further ischaemic damages [142].

Although outside of the scopes of the current review, it should be emphasized that cerebrovascular heterogeneity should not be neglected. Significant regional, cellular, and functional differences exist and should be taken into account with specific cerebrovascular regions being differentially implicated in the pathophysiology of various neurological processes [9, 11, 143–145].

## Conclusions

To conclude, experimental and clinical studies strongly suggest a perceptible and complex crosstalk between the lung and the brain in patients with acute lung injury. The aim of the current review was to focus on the pathophysiology of acute brain injury in patients with ALI/ARDS. As we have shown, ARDS involves activation of systemic inflammatory cascades and neuroinflammation, so that the brain of ARDS patients and, especially, the hippocampus becomes very vulnerable to the development of secondary lung-mediated acute brain injury. A protective ventilator strategy could reduce or even minimize further systemic release of inflammatory mediators and, thus, maintain brain homeostasis. Further refinements are needed to enhance our understanding of ARDS associated acute brain injury and evaluate optimal management of lung-associated acute brain injury.

## Data Availability

Not applicable.
